# Anatomic locations of ureterovascular fistulae: a review of 532 patients in the literature and a new series of 8 patients

**DOI:** 10.1186/s42155-024-00475-1

**Published:** 2024-08-14

**Authors:** Mohammed Shamseldin, Hendrik Heers, Thomas Steiner, Ralf Puls

**Affiliations:** 1 Department of Radiology, Helios Klinikum Erfurt, Nordhäuser Str. 74, Erfurt, 99089 Thuringia Germany; 2 Department of Urology, Universitätsklinikum Gießen Und Marburg, Marburg, Germany; 3 Department of Urology, Helios Klinikum Erfurt, Erfurt, Thuringia Germany

## Abstract

**Introduction:**

Ureterovascular fistula (UVF) is a rare but potentially life-threatening condition. Since its primary description by Moschkowitz in 1908, many case reports, studies and reviews have been written about this condition with the suggestive symptoms and risk factors repeatedly discussed. This study will be focusing on the different locations of 532 out of 605 fistulae published from 1908 up to 2022 besides eight new patients of our own.

**Material and methods:**

A systematic review of the literature started using PubMed database searching for “ureteroarterial fistula”, “arteriovascular fistula” and “uretero vascular fistula” was performed yielding 122, 62 and 188 results respectively. Those studies and the cited literature in each study were examined to include studies, which did not appear in the primary search. A total of 605 patients in 315 publications were gathered. Only studies mentioning new patients, a clear indication of the location of the UVF, the presence/absence of urinary diversion (UD) as well as the type of UD if present were included. Ten duplicates as well as studies lacking information regarding the UVF and/or the UD (seven publications with 63 patients) were excluded, with 298 publications including 532 external patients remaining. Eight internal cases were included with a total of 540 cases.

**Results:**

From the 540 included cases, 384 patients (71.1%) had no UD compared to 156 patients (28.9%) with UD. Due to the anatomical ureteral course, the common iliac artery (CIA) was the most common vascular component of UVF, irrespective of the presence or absence of UD. Any dispute to whether the crossing point is the common or the external iliac artery (EIA) was settled for the CIA. Further common vascular components besides CIA include the aorta, EIA, internal iliac artery (IIA) including its branches and vascular bypasses including the anastomosis sites. Other unusual arterial localizations were stated under the “others” category.

**Conclusion:**

Identifying the location of the bleeding artery in UVF is critical and represents the most important step for successful management. We present the largest summary of described locations up to date including our own.

**Graphical Abstract:**

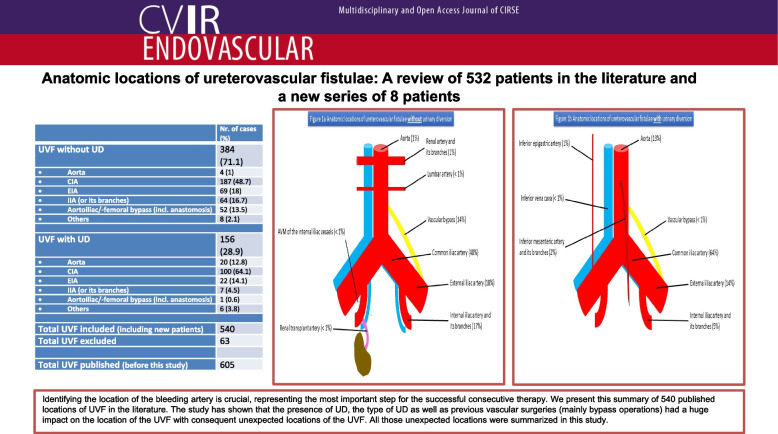

## Introduction

In the literature usually referred to as ureteroarterial fistula or arterioureteral fistula (UAF/AUF) is a potentially life-threatening condition which should be abruptly and correctly diagnosed and treated. The term ureterovascular fistula (UVF) would be used in this article instead of the more commonly used ureteroarterial fistula to emphasise the possiblity of venous participation in ureteral fistulae. The condition was primarily described by Moschkowitz in 1908 [1], UVF could be classified into primary and secondary UVF based on the underlying pathology. The most common and serious symptom is macrohematuria [2–4]. The majority of the cases are secondary (85%) due to previous pelvic interventions including pelvic surgery to remove pelvic tumors (89%) with combined radiation (67%) being an additional risk factor. The most common risk factor is the presence of a chronic ureteral stent in 73.7% of the cases [3]. Primary UVF are rare (15%) and involve primarily a vascular pathology such as aneurysms, vascular malformations and aberrant vessels [5, 6]. In the case of uretero-caval fistula in which the inferior vena cava was involved as the vascular (venous) component, the risk factors are quite similar, however without hematuria present, but rather nonspecific clinical signs such as fever with pulmonary microembolism due to formation of a thrombus at the vascular point of the fistula [7].

Therapeutic options include historically surgical treatment with or without transarterial embolization [8–10]. With the rapid advancement of the minimally invasive endovascular therapy especially of stent grafts (SG) and embolic materials, this became quickly the primary option in many centers, both favored by both patients and medical staff, taking into consideration that the majority are cancer patients suffering from multi-morbidities, which make surgery quite difficult.

Irrespective of the underlying pathology, the single most important key to a successful treatment of UVF is to accurately identify the vascular component sharing in the fistula. To reach that goal, an accurate patient´s history should be documented. Important is get a detailed history of previous operations in the abdominopelvic region, whether the patient was subjected to radio- and/or chemotherapy, the presence of a urinary diversion (UD) and the type of diversion which was performed. Urinary diversion is a surgical procedure performed when the bladder is not functioning properly or has been removed due to disease or injury that reroutes the normal flow of urine from the kidneys and ureters to an alternative exit route. The two main categories of UD are urostomy (eg. Ileal conduit and cutaneous ureterostomy) and continent urinary diversion (eg. Neobladder). This is specifically critical due to the anatomical changes regarding the course of the ureter(s) leading to a high risk of unexpected unusual locations of the UVF involving vascular components, which are not normally in direct proximity to the ureters.

In our review, we will be presenting a detailed description of all published UVF locations in the literature through reviewing 315 published articles. We added eight cases from our institute with important teaching points included in the discussion section. The novelty of this article is to indicate all published atypical locations of UVF, especially in cases with UD, which were mainly scattered in the literature in form of case reports due to the rarity of the condition.

## Material and methods

**Fig. 1:**
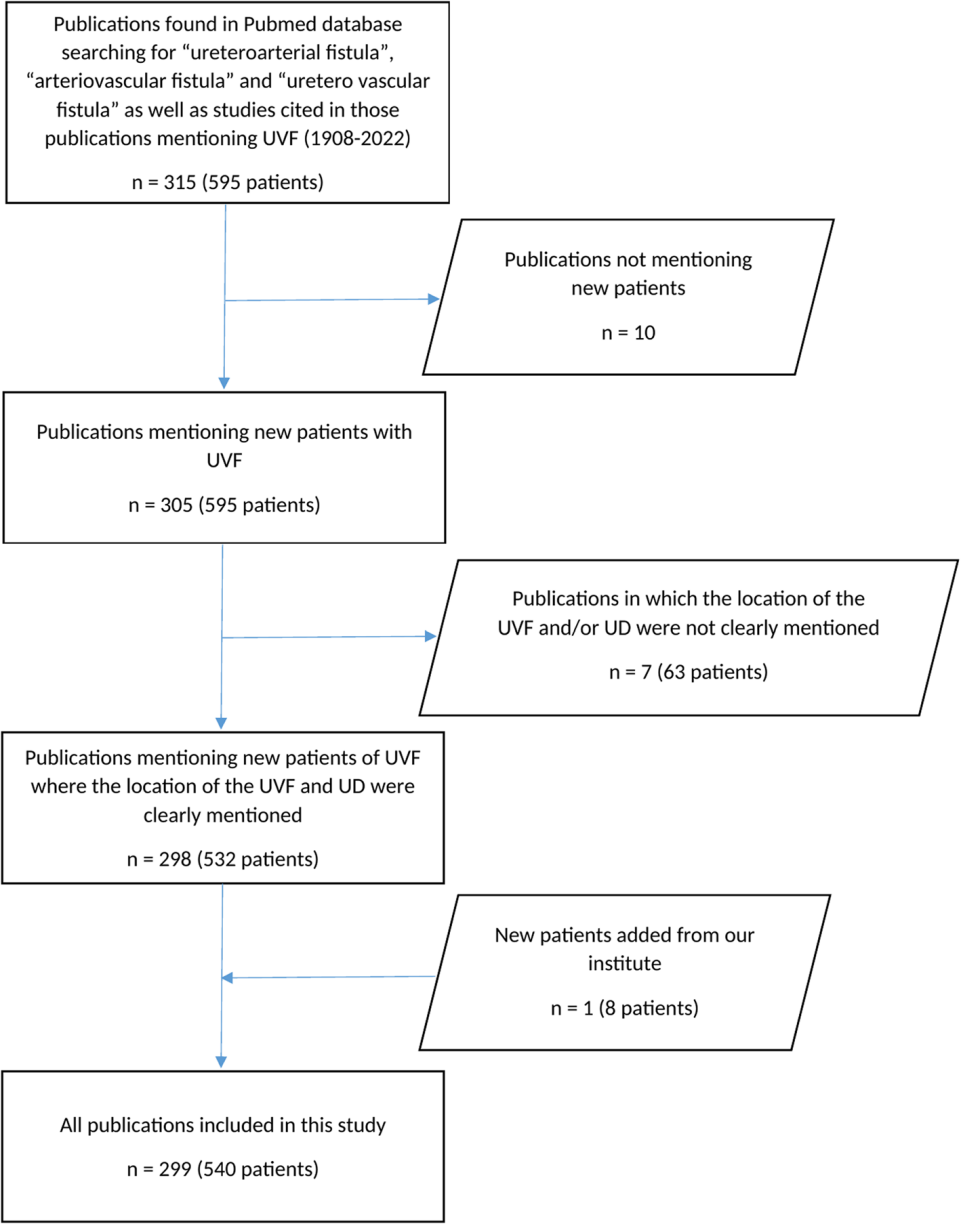
Publications search and investigations with exclusion criteria

Our systematic review of the literature started using PubMed database searching for “ureteroarterial fistula” “arteriovascular fistula” and “uretero vascular fistula” was performed yielding 122, 62 and 188 results respectively from 1908 up to 2022. All the studies found were examined and then all the literature references cited in each study were separately examined to gather the largest possible number of published articles including studies, which did not appear in the primary search in the PubMed database. A total of 605 patients were found in a total of 315 publications. Each study was then examined to determine whether new patients were included, a clear indication of the location of the UVF, the presence/absence of UD as well as the type of UD if present. Ten duplicates not including new patients were excluded including only original articles with newly mentioned cases. The lack of sufficient information regarding the UVF and/or the UD as mentioned above was used to exclude seven further publications including 63 patients, with 298 publications including 532 external patients remaining. After the addition of eight cases of our own, a total of 540 patients were included in this study (Fig. 1).

The patients from our institute were referred to us from the department of urology with a suspected UVF for endovascular therapy between February 2017 and March 2021 (Table 1). A detailed overview of all the publications considered as well as the vascular and ureteral components of UVF in each study were gathered and supplied as supplementary files. In our hospital, the side of the bleeding was always identified using endoscopy, usually during an exchange of ureteral stents. A biphasic Computer tomography (CT) scan with arterial and portal venous phases was perfomed in all patients which did not have any CT scans in our system prior to the onset of symptoms and had no UD (six out of eight patients). Older CT scans of the remaining two patients were used to identify the possible vascular component of the fistula. The aim of the CT scan was mainly to identify the suspected vessel involved in the fistula whether through vascular changes such as pseudoaneuryms and vascular irregularities or through proximity to the ureter on the bleeding side identified during endoscopy. A digital subtraction angiography (DSA) was then routinely performed to treat the patient. A standard right femoral access using a 5-French (F) Destination guiding sheath (Terumo, Tokyo, Japan) and a 4F-UF-flush catheter (Cordis, California, USA) or 4F-Sidewinder Simmons 1 catheter (Cordis, California, USA) in the case of the IMA were performed. A trial to disclose the UVF with and without applying a provocative maneuver through temporarily removing the ureteric catheter and applying contrast medium at the point of ureteral crossing was attempted in all cases. In some cases, the only way to visualize the UVF was a selective injection of contrast medium in the direct proximity of the suspected crossing point after temporary removal of the ureteral stent through the urologist in the angiography suite as a provocation.

**Table 1:** Internal patients with UVF in Helios Klinikum Erfurt

Case	Age (years)	Gender	Primary disease and therapy	Risk Factors of UAF	Symptoms	Radiological diagnosis	UAF visualized	UAF location	UAF side	Urinary diversion	UAF treatment	Technical success	Clinical success	Postinterventional medication
1	71	M	- Rectal cancer resection + radiation therapy + chemotherapy - Prostate cancer TURP^a^	- Radiation therapy - Chemo-therapy - ureteric catheter (obstructive BPH^b^) - recurrent urosepsis - Phenprocoumon anticoagulation (absolute tachyarrhythmia + atrial fibrillation)	- recurrent Macro-hematuria with bladder tamponade	- ureteroscopy bleeding from left ostium - CT negative + ureteroarterial crossing - overview DSA negative (2x) - selective DSA positive	Yes (direct in selective DSA)	IIA	L	No	- Coiling + Glue embilzation IIA - Viabahn Stent	Yes	Yes	- Phenprocoumon pause for 6 weeks - ASA^c^ 100 mg for 6 weeks - LMWH^d^ 20 mg s.c. for 6 weeks
2	80	F	- Rectal cancer resection + radiation therapy + chemotherapy	- Radiation therapy - Chemo-therapy - ureteric catheter (post radiogenic stricture) - recurrent urosepsis - ASA^c^ + Clopidogrel dual platelet inhibition (TVCAD^e^ + coronary stents) - recurrent Urosepsis	- recurrent Macro-hematuria with bladder tamponade - acute pain of the left flank	- ureteroscopy bleeding from left ostium - overview DSA negative (1x) - selective DSA positive	Yes (direct in selective DSA)	IIA branch (SVA^f^)	L	No	- Glue embolization SVA^f^ - Coiling IIA - Viabahn Stent	Yes	Yes	- ASA^c^ 100 mg lifelong - Clopidogrel 75 mg long term
3	84	M	- Urothelial carcinoma of the left ureter distally Laser ablation	- Laser ablation of the left ureter - ureteric catheter (malignant ureteric obstruction) - recurrent urosepsis - Clopidogrel monotherapy (PAD^g^ + coronary stent + ischemic cardiomyopathy)	- recurrent Macro-hematuria	- ureteroscopy bleeding from left ostium - overview DSA negative - selective DSA negative	No	IIA branch (IVA^h^)	L	No	- Particle embolization IVA^h^ - Coiling IIA - Advanta V12	Yes	Yes	- Clopidogrel 75 mg lifelong - ASA^c^ 100 mg for 4 weeks
4	79	F	- Rectal cancer resection + radiation therapy + chemotherapy	- Radiation therapy - Chemo-therapy - ureteric catheter (post radiogenic stricture) - ASA^c^ monotherapy (ischemic cardiomyopathy)	- recurrent Macro-hematuria	- ureteroscopy bleeding from left ostium - CT negative + ureteroarterial crossing - overview DSA negative (2x) - selective DSA negative	No	IIA	L	No	- Coiling IIA - Viabahn Stent	Yes	Yes	- ASA^c^ 100 mg lifelong
5	76	M	- Prostate cancer TURP^a^ + radiation therapy	- Radiation therapy - ureteric catheter (post radiogenic stricture)	- recurrent Macro-hematuria with bladder tamponade	- ureteroscopy bleeding from right ostium - R-UPG^i^ active bleeding into the retroperitoneal space - CT pseudoaneurysm + retroperitoneal contrast medium from the R-UPG^i^ + ureteroarterial crossing - DSA pseudoaneurysm	Yes (indirect through pseudo-aneurysm + active bleeding in R-UPG^i^ and CT)	EIA	R	No	- Vascular plug IIA - Advanta V12 Stents (3x)	Yes	Yes	- ASA^c^ 100 mg lifelong
6	85	M	- Rectal cancer resection + radiation therapy + chemotherapy	- Radiation therapy - Chemo-therapy - ureteric catheter (post radiogenic stricture) - ASA^c^ monotherapy (ischemic cardiomyopathy)	- recurrent Macro-hematuria	- ureteroscopy bleeding from left ostium - selective DSA negative - CT negative + ureteroarterial crossing	No	CIA	L	No	- Coiling IIA + covering IIA using stent graft (during EVAR)	Yes	Yes	- ASA^c^ 100 mg lifelong
First recurrence after 3 years				- Eliquis anticoagulation (atrial fibrillation)	- recurrent Macro-hematuria with bladder tamponade - acute pain of the left flank	- CT pseudoaneurysm (at the distal end of the stent graft) - overview DSA pseudoaneurysma	Yes (pseudo-aneurysm in CT and DSA)				Viabahn VBX + postdilatation	Yes	No	- Eliquis pause for 1 weeks - LMWH^d^ 20 mg s.c. for 1 week - ASA^c^ 100 mg lifelong
Second recurrence after 24 h						- overview DSA pseudoaneurysma					postdilatation	Yes	Yes	
7	77	M	- Rectal cancer resection - Prostate cancer radical prostatectomy + pelvic lymphadenectomy + postoperative radiation + LHRH analogs^j^)	- Radiation therapy - ureteric catheter (iatrogenic injury of the distal ureter and urinary bladder cystectomy and ileum-conduit on the right side) - Enoxaparin (chronic immobility due to tetraplegia)	- recurrent Macro-hematuria	- CT negative + ureteroarterial crossing - selective DSA negative	No	EIA	R	Yes	- Coiling IIA - Viabahn Stents (2x)	Yes	Yes	- Enoxaparin 30 mg
8	82	M	- Prostate cancer radical prostatectomy + postoperative radiation + LHRH analogs^j^)	- Radiation therapy - ureteric catheter (recurrent post radiogenic and postinfectious vesicointestinal fistulas cystectomy + ureterocutaneostomy on the right side)	- recurrent Macro-hematuria - acute pain of the left flank	- R-UPG^i^ positive - CTA (2 months ago) ureteroarterial crossing - overview DSA negative - selective DSA with provocation positive	Yes (direct in R-UPG^i^ and selective DSA with provocation)	IMA	-	Yes	Front door – back door coil embolization IMA	Yes	Yes	-

^a^*TURP* Transurethral resection of the prostate

^b^*BPH* Benign prostatic hyperplasia

^c^*ASA* Acetylsalicylic acid

^d^*LMWH* Low-molecular-weight heparin

^e^*TVCAD* Triple vessel coronary artery disease

^f^*SVA* Superior vesical artery

^g^*PAD* Peripheral artery disease

^h^*IVA* Inferior vesical artery

^i^*R-UPG* Retrograde ureteropyelography

^j^*LHRH* analogs Luteinizing hormone-releasing hormone analogs

In all cases, therapy was performed either through embolization of the vessel in question whenever possible, for example the internal iliac artery (IIA) or the inferior mesenteric artery (IMA) using coils or a vascular plug or by using particle embolization in the case of inferior vesical artery (IVA) or glue embolization in the case of superior vesical artery (SVA). In cases where the common iliac artery (CIA), external iliac artery (EIA) or IIA were involved, a slightly oversized stent graft (1 mm) was used to cover the UVF from the arterial side or covering the origin of the IIA. As long as the origin of the IIA was expected to be covered by the stent graft, an additional proximal embolization of the IIA using coils or a vascular plug was routinely performed to disallow any possible collateralization.

## Results

In this study, we were able to collect the largest number of published UVF based on their location. The localization of the UVF was the single most important step in managing the condition. We thereby present all the published locations of UVF in the literature up to date (Table 2). From the 540 included cases, 384 patients (71.1%) had no UD compared to 156 patients (28.9%) with UD (Table 2). Since the ureters anatomically cross the pelvic arteries over the distal CIA directly above the iliac bifurcation, the CIA was the most common vascular component of UVF, irrespective of the presence or absence of UD. Any dispute to whether the crossing point is the CIA or the EIA was settled for the CIA. Further common vascular components besides CIA include the aorta, EIA, IIA including its branches and vascular bypasses including the anastomosis sites. Other unusual arterial localizations were stated under the “others” category.

**Table 2:** Summary of UVF cases according to localization

			Nr. of cases (%)	
**UVF without UD**			**384 (71.1)**	
• Aorta			4 (1)	
• CIA			187 (48.7)	
• EIA			69 (18)	
• IIA (or its branches)			64 (16.7)	
• Aortoiliac/-femoral bypass (incl. anastomosis)			52 (13.5)	
• Others			8 (2.1)	
**UVF with UD**			**156 (28.9)**	
• Aorta			20 (12.8)	
• CIA			100 (64.1)	
• EIA			22 (14.1)	
• IIA (or its branches)			7 (4.5)	
• Aortoiliac/-femoral bypass (incl. anastomosis)			1 (0.6)	
• Others			6 (3.8)	
**Total UVF included** *(including new patients)*			**540**	
**Total UVF excluded**			**63**	
**Total UVF published** *(before this study)*			**605**	

We further subcategorized the locations according to the presence and type of UD, as a distorted course of the ureters was the main reason for unexpected localizations of UVF.

Rare UVF including unusual vascular involvement, unusual urinary tract involvement, double-vessel UVF and complex UVF were recollected in separate tables (Tables 3, 4, 5 and 6).

**Table 3:** List of rare and unexpected UVF locations in patients without UD

Vessel involved	**Urinary tract involved**	**Study number**	**Study name**
1- Lower pole segmental renal artery	Ureter	11	Castle et al
2- EIA	Ureter of a failed atrophic kidney transplant	15	Geevarghese et al
3- Intrarenal segmental artery	Ureter	18	Augustin et al
4- EIA	Urinary bladder	38	Nakai et al
5- Artery stump of a failed renal transplant	Ureter	76	List et al
6- AVM of the internal iliac vessels	Ureter	138	Sharma et al
7- Ureteral branch of a renal artery	Ureter	156	Siablis et al
8- CIA Stump	Ureteral stump after nephrectomy	157	Noh et al
9- IIA	Ureteral stump after nephrectomy	119	Goldberg et al
10- CIA	Ureteral stump after nephrectomy	132	Pozzilli et al
11- Retrograde filling of the non-functional CIA with CIA aneurysm after aorto-bifemoral graft due to AAA	Ureter	164	Van Damme et al
12- Lower pole segmental renal artery	Ureter	174	Wagner et al
13- Iliac anastomosis of an aorto-biiliac bypass	Ureteral stump after nephrectomy	185	Tijunaitis et al
14- CIA	Ureteral stump after nephrectomy	190	Kibrik et al
15- Non-functional occluded right limb of an aorto- right iliac - left femoral bypass graft	Ureter	202	Wheatly et al
16- Right limb of an inverted Dacron graft prosthesis	Ureteral stump after nephrectomy	229	Ferretti et al
17- Renal artery pseudoaneurysm of a renal transplant	Ureter	236	Turunc et al
18- CIA	Ureteral stump after nephrectomy	246	Baum et al
19- CIA	Ureteral stump after nephrectomy	249	Schulz et al
20- Fourth right lumbar artery (L4)	Ureter	265	Chen et al
21- Aortic anastomosis of a left aortofemoral bypass	Ureteral stump after nephrectomy	291	Mironiuc et al
22- CIA	Ureteral stump after nephrectomy	306	Hodges et al
23- Unidentified branch of the IIA	Urinary bladder	307	Nicita et al
24- Iliac anastomosis of a right iliofemoral autogenous vein extra-anatomic graft	Urinary bladder	308	Jaha et al

**Table 4:** List of rare and unexpected UVF locations in patients with UD

Vessel involved	Urinary tract involved	Urinary diversion	Study number	Study name
1- EIA	Ileal conduit	Ileal conduit	106	Beaugie et al
2- Inferior mesenteric artery (IMA)	Ureter	cutaneous double-barrel ureterostomy	124	Dervanian et al
3- Aortic stump after excision of the CIA with femoro-femoral bypass	Ureter	right cutaneous ureterostomy	149	Ishibashi et al
4- Aorta and CIA	Ileal conduit	Ileal conduit	157	Noh et al
5- EIA	Ileal conduit	Ileal conduit	217	Gómez et al.
6- Superior rectal artery (branch of the IMA)	Ileal conduit	Ileal conduit	266	Altaha et al
7- CIA	Ileal conduit	Ileal conduit	267	Coello Tora et al
8- EIA	Ileal conduit	Ileal conduit	274	Sekito et al
9- EIA	Ileal conduit	Ileal conduit	275	Beaugie et al
10- CIA	Ileal conduit	Ileal conduit	276	Hindmarch et al
11- Aorta	Ileal conduit	Ileal conduit	277	Ishibashi et al
12- EIA	Ileal conduit	Ileal conduit	278	Sasaki et al
13- EIA	Ileal conduit	Ileal conduit	279	Castillo et al
14- EIA	Ileal conduit	Ileal conduit	280	Sukha et al
15- EIA	Ileal conduit	Ileal conduit	281	Morlacco and Zattoni et al
16- Inferior vena cava	Ureter	Bricker ileal conduit	290	Pultrone et al
17- CIA	IP Neobladder	Orthotopic neobladder	292	Tatsuishi et al
18- right inferior epigastric artery	Ureter	bilateral cutaneous ureterostomy	312	Nakama et al
19- left inferior epigastric artery	Ureter	left cutaneous ureterostomy	313	Fujinama et al
20- Inferior mesenteric artery (IMA)	Ureter	Cutaneous ureterostomy	316	Shamseldin et al

**Table 5:** List of Double vessel UVF

Study number	Study name	Vessel Fistula 1	Urinary tract Fistula 1	Vessel Fistula 2	Urinary tract Fistula 2	Urinary diversion
19	Moon et al	EIA	Ureter	IIA	Ureter	-
97	Feuer et al	CIA	Ureter	EIA	Ureter	Orthotopic bladder substitution
124	Dervanian et al	CIA	Ureter	IMA	Ureter	Bilateral, cutaneous double-barrel ureterostomy
227	Han et al	CIA	Ureter	EIA	Ureter	Orthotopic bladder substitution

**Table 6:** List of complex UVF

Study number	Study name	Complex UVF	Urinary diversion
40	Diner et al	Ureteric-Vascular (CIA)-Enteric (Hartman´s pouch) fistula	-
48	Morgan et al	Ureteric-Vascular (CIA)-Enteric (terminal ileum) fistula	-
57	Joglekar et al	Ureteric-Vascular-Cutaneous fistula	-
198	Kurata et al	Ureteric-Vascular (IIA)-Colonic (Rectum) fistula	-
230	Policha et al	Ureteric-Vascular (distal anastomosis of a right interposition CIA Dacron graft bypass)-Colonic (Caecum) fistula	-
243	Abdul Rashid et al	Ureteric-Vascular (EIA)-Enteric (small bowel) fistula	-
289	Amahzoune et al	Ureteric-Vascular (IIA)-Colonic (Caecum) fistula	-

**Fig. 2:**
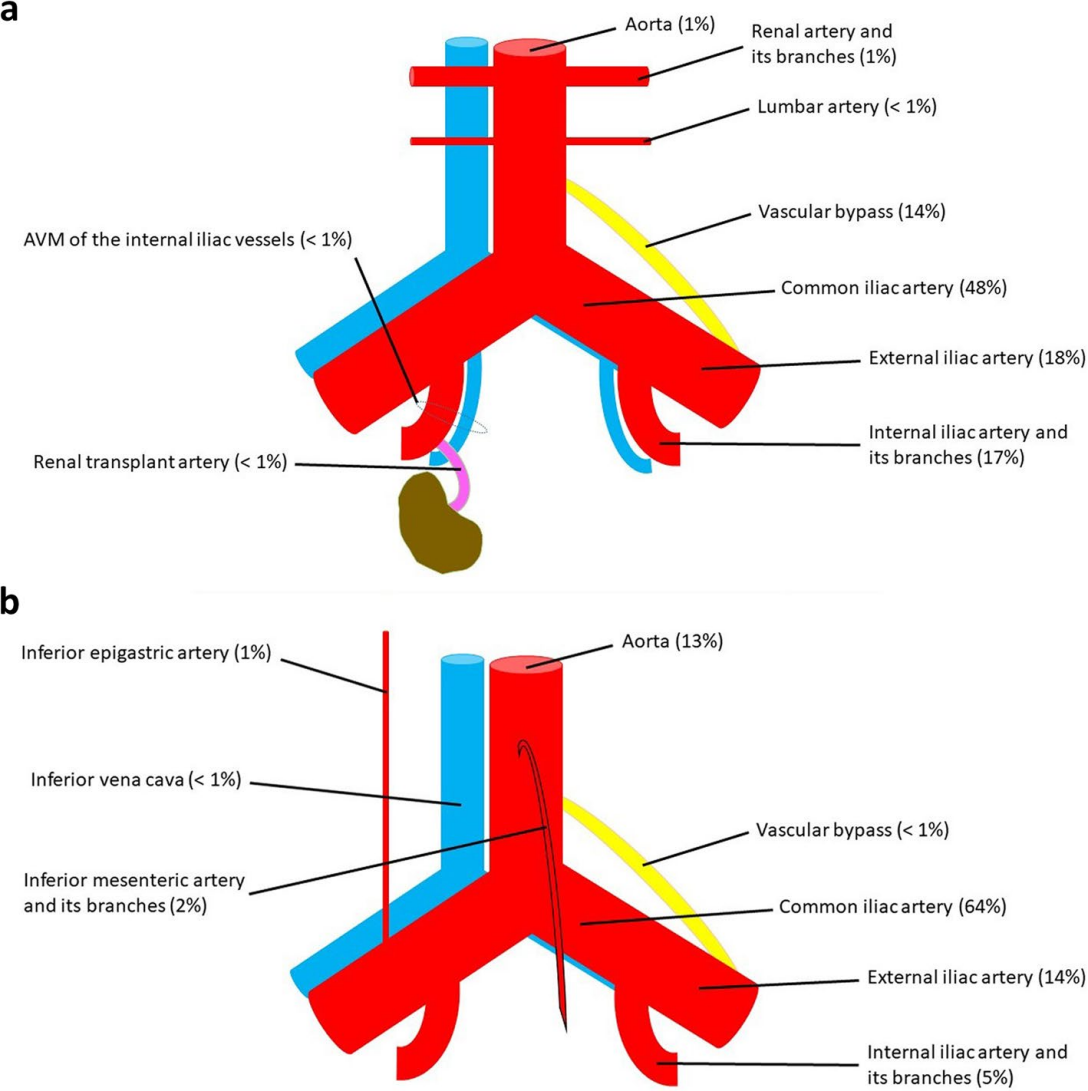
**a** Anatomic locations of ureterovascular fistulae without urinary diversion. **b** Anatomic locations of ureterovascular fistulae with urinary diversion

A summary of the most common locations of UVF with and without UD are shown in Fig. 2 [11]. Although the most common sites of UVF with or without UD were the CIA (64% and 48% respectively) and EIA (14% and 18% respectively), the presence of UD increased the incidence of the aortic involvement drastically up to 13% (only 1% without UD). This is supposedly due to the medial para-aortal course of the ureters due to the UD. If the UD cross the midline, the IMA as a midline artery should be considered as a possible vascular component of the UVF.

## Discussion

Ureterovascular fistula (UVF) is an abnormal communication between a vascular structure (artery, vascular bypass or rarely a vein) and the ureter. It is a rare condition with a few more than 600 cases in total published in the literature. It is a potentially life threatening condition leading to intermittent but usually massive bleeding. Mortality is estimated to be approximately 9% and morbidity is 23% [10, 12].

UVF could be classified into primary and secondary UVF based on the underlying pathology. The majority of the cases are secondary (85%) due to previous pelvic interventions including pelvic surgery to remove pelvic tumors (89%) with combined radiation (67%) being an additional risk factor. The most common risk factor is the presence of a chronic ureteral stent in 73.7% of the cases according to Das et al. triggering a chronic mechanical erosion between the continuously pulsating artery or nearby vessel and the stented ureter, triggering a local inflammatory response and eventual necrosis at contact point between the vessel and the ureter [3]. All our eight patients (100%) had chronic ureteral stents supporting the contribution of this risk factor to the formation of UVF. Other risk factors include previous pelvic surgery and radiotherapy [9, 12, 13]. Primary UVF are rare (15%) and involve primarily a vascular pathology such as aneurysms, vascular malformations and aberrant vessels [5].

The occurrence of unexplained gross hematuria with variable clot passage and flank pain in a patient with a history of pelvic surgery, a chronic ureteral stent, and/or history of pelvic radiotherapy is highly suspicious for a UVF [9, 14]. It is important to bear in mind that the source of a urinary tract bleeding through the ureteral orifice is not always the kidney. If the urologist and interventional radiologist do not keep that in mind, this could lead to unnecessary renal embolization or even nephrectomy due to misdiagnosis [6, 12, 15]. A high degree of suspicion in patients with known risk factors for UVF is the key to a correct diagnosis.

In our hospital, sufficient history would be taken as well as clinical urological examination of the patient excluding other more common cases of macrohematuria such as trauma, recent renal surgery, kidney stones, renal tumor and urinary tract infection. If based on history and examination, UVF was suspected, the side of the bleeding was always initially identified using endoscopy. This step is crucial to help minimize the planning time of the procedure through focusing only on the course of the bleeding ureter during examination of the subsequent CT scan. During angiography, the sole examination of the bleeding side instead of wasting time on the normal side would also minimize the intraprocedural time, thereby reducing the time to stop the bleeding and the radiation exposure. Lock et al. [16] were able to show that there was no significant difference in the location when it comes to the side of the UVF. Stating the side of the fistula’s location was in most cases irrelevant, especially in cases where no UD was present, and therefore the side (right vs. left) was not considered in our review. We mentioned the side in our new patients in case future studies prove otherwise.

CT scan was helpful according to the literature in only 48% of the cases and ureteropyelography in 52% [17] to directly visualize the UVF. We performed a biphasic CT scan with arterial and portal venous phases was perfomed in six out of our eight patients. The aim of the CT scan was mainly to identify the suspected vessel involved in the fistula whether through vascular changes such as pseudoaneuryms and vascular irregularities or through proximity to the ureter on the bleeding side identified during endoscopy. The remaining two patients had older CT scans where the suspected vessel could be identified. A direct visualization of the bleeding through the fistula between the vessel and the catheter bearing ureter is often not possible to see on cross sectional imaging [18] mainly due to the catheter usually closing the fistulous opening from the ureteral side as well as the difficult distinction of contrast medium due to the intraureteral catheter. Only two of the six patients showed direct signs of vascular injury in the CT scan contributing to 33% of the cases.

Direct signs like vessel irregularities, compression and pseudoaneurym at the site of the ureteral crossing are clear evidence of the site of vascular involvement, however quite uncommon [12, 19]. Even in the DSA with or without a provocative maneuver—as a gold standard for diagnosis—was only successful in 62% of the cases [17]. However, a close relation between the ureter and a nearby vessel per se in the cross sectional imaging was in many cases enough to locate the UVF indirectly. Due to the intermittent bleeding nature of the UVF and the intraureteral catheter closing the ureteral side of the UVF, an active bleeding during the point of performing an angiography is not always possible. Therefore, even if the UVF was not directly visualized, the point of ureterovascular proximity should be managed as the bleeding point.

**Fig. 3:**
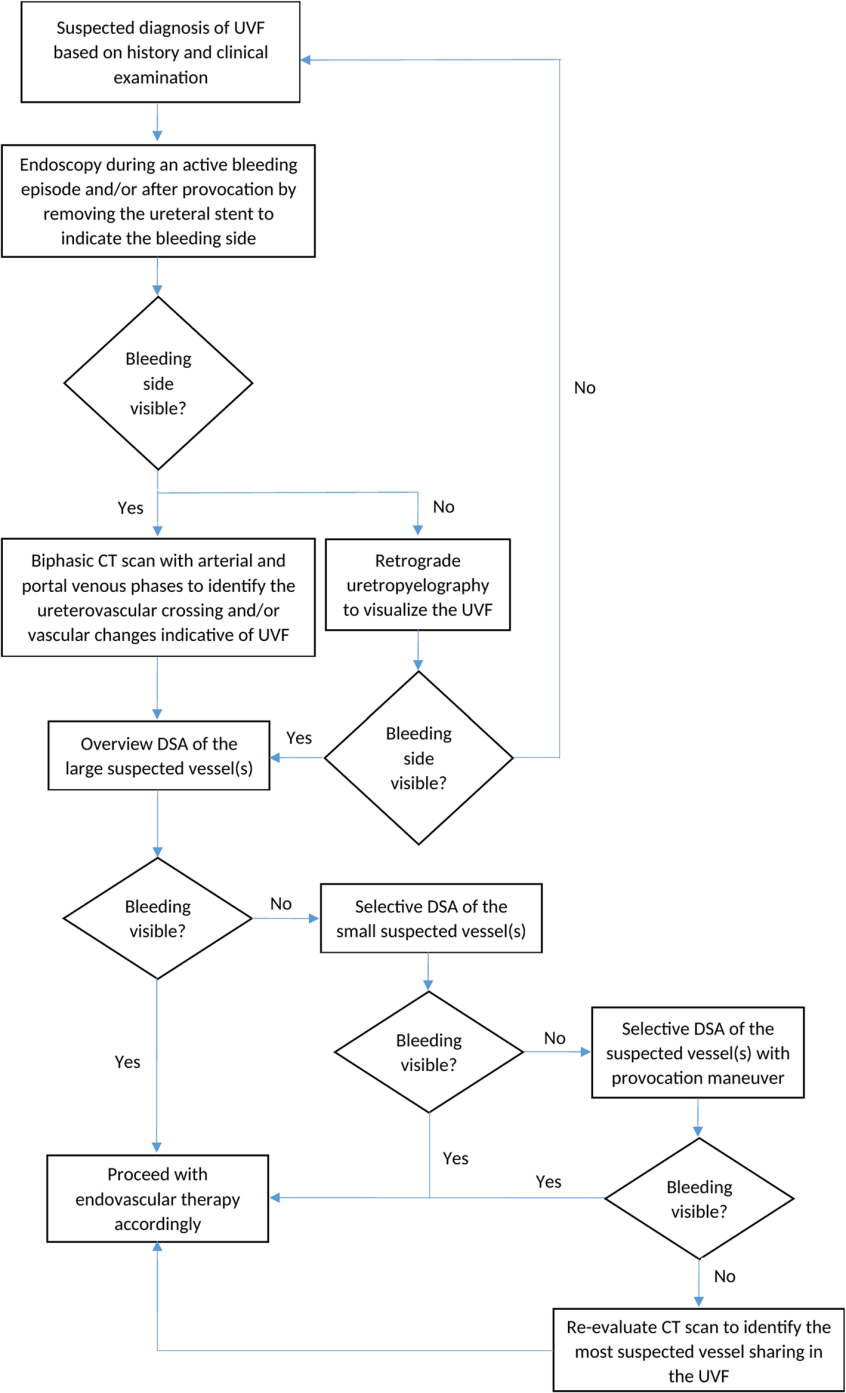
Recommended algorithm of the workflow to diagnose UVF

Based on the above steps, a standard approach to localize suspected UVF in our institute was constructed (Fig. 3). The side of the bleeding was initially identified using endoscopy, usually during an exchange of ureteral stents. Identifying the suspected artery was performed using a contrast medium biphasic CT scan with arterial and portal venous phases. An angiography was then routinely performed through a right femoral access to try to disclose the UVF with and without applying a provocative maneuver through temporarily removing the ureteric catheter and applying contrast medium at the point of ureteral crossing. In some cases, the only way to visualize the UVF was a selective injection of contrast medium in the direct proximity of the suspected crossing point. If the UVF is still not visible, a re-evaluation of the CT scan to determine the most suspected vessel is recommended before proceeding with endovascular therapy.

Historically, different treatment options including surgery or surgery combined with transarterial embolization have been described. More recently, SG placement has been reported as an effective alternative treatment [20]. Considering the absence of treatment guidelines, there is a clear trend that the endovascular therapy of UVF is preferred due to the minimally invasive nature of the procedure when compared to surgical therapy in multi-morbid patients. However, multicentric prospective studies are needed in order to provide strong, evidenced-based recommendations and treatment guidelines.

The endovascular treatment of UVF involving the CIA and EIA is quite similar necessitating covering the ureteral crossing using a SG. In case the SG would be covering the origin of the IIA, a coil embolization of the IIA should be performed prior to the embolization to avoid recurrence through retrograde perfusion from the gluteal arteries (Patients 6 and 7, Table 1). An alternative to coils would be using a vascular plug (Patient 5, Table 1). If the source of the bleeding is the main stem of the IIA or one of its two main branches, namely the anterior or posterior trunk, a front door – back door embolization of the bleeding point should be sufficient (Patients 1, Table 1) or in combination with another embolization agent like glue eg. 1:1 mixture of N-Butyl Cyanoacrylate Glue and Lipiodol (Patient 1, Table 1). However, if a more distal branch of the IIA is involved, a more distally reaching embolization agent is recommended like glue eg. 1:4 mixture of N-Butyl Cyanoacrylate Glue and Lipiodol or particles eg. Embospheres between 100 and 500 µm (Patients 2 and 3, Table 1). But due to the potentially life-threatening nature of the condition, all patients involving the IIA or any of its branches was aggressively managed with successive coiling of the IIA and/or covering the origin of the IIA using a SG (Patients 1–4, Table 1). Other unusual locations of UVF should be treated correspondingly. In our case, where the IMA was involved, a front door – back door coil embolization was necessary (Patient 8, Table 1). Below, two of the cases are further discussed.

**Fig. 4:**
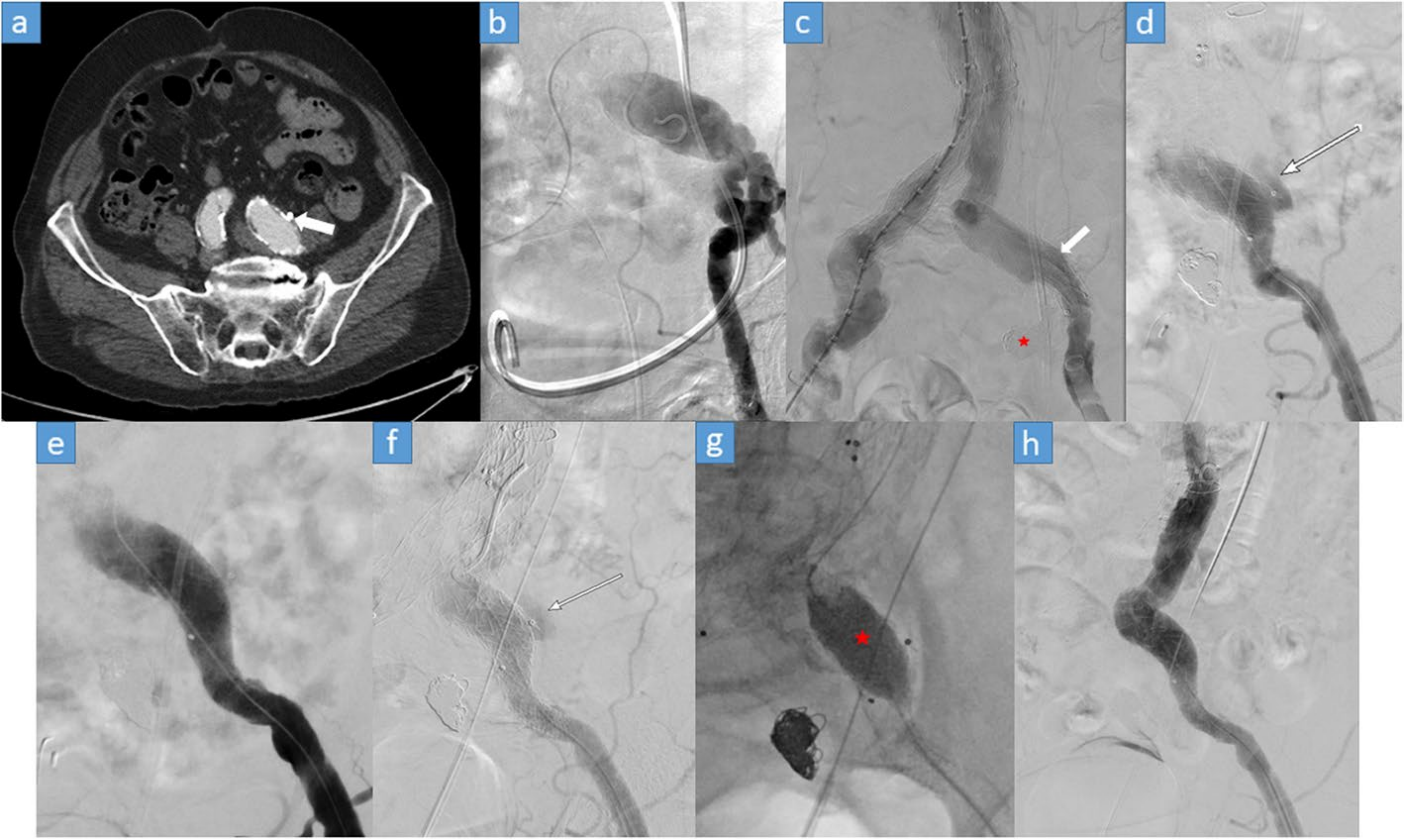
**a** CT scan showing the point of vascular crossing over the distal CIA with a ureteral stent seen (arrow). **b** A negative DSA shortly prior to a planned endovascular aortic repair (EVAR) due to an aortic aneurysm. During this angiography session, a kidney bleeding was also excluded (not seen). **c** Coiling of the IIA (star) and coverage of the origin of the IIA using the left leg of the SG (arrow) were performed during the EVAR procedure. **d** Pseudoaneurysm at the distal end of the stent graft denoting the first recurrence after 3 years (arrow). **e** Distal elongation of the SG using a Viabahn VBX SG with complete closure of the pseudoaneurysm in the immediate control image. **f** DSA after 24 h due to recurrent macrohematuria showing recurrence of the pseudoaneurysm (arrow). **g** Post-dilatation of the Viabahn VBX SG using a larger balloon (star) at the site of the visible pseudoaneurysm. **h** Complete obliteration of the pseudoaneurysm

An interesting patient was patient 6 who suffered from rectal cancer and underwent rectal resection, radiation therapy and chemotherapy. The patient received a ureteric stent due to a post radiogenic stricture and was receiving acetylsalicylic acid and Apixaban due to ischemic cardiomyopathy. The initial UVF of the CIA was managed successfully for three years through a simultaneous treatment of an abdominal aortic aneurysm through the iliac leg graft and coiling of the IIA (Fig. 4a, b, c). However due to the known risk factors in addition to new anticoagulation therapy for atrial fibrillation, a recurrence at the distal end of the SG took place with a visible pseudoaneurysm in the DSA (Fig. 4d). This was managed by elongating the SG distally (Fig. 4e). Due to the massive improvement of the material used in the field of interventional radiology, we used the balloon expandable VBX stent grafts (Gore, Delaware, USA) which are lightly remodeled using larger balloons reaching much higher diameters in case the obstruction of the UVF was not enough due to elongation or segmental ectasia or aneurysm of the vessel. A new recurrence this time occurred within 24 h. The DSA revealed a persistent pseudoaneurysm which was successfully managed by sufficient postdilatation of the VBX stent graft (Fig. 4f, g, h). The uniqueness of this case is the multiple recurrences of the bleeding due to an insufficient initial therapy and how the development of endovascular material such as VBX stent grafts allow simple post-dilatation of a pre-existing SG to reach the desired diameter and block the UVF in case of a primarily undersized stenting of the vessel.

**Fig. 5:**
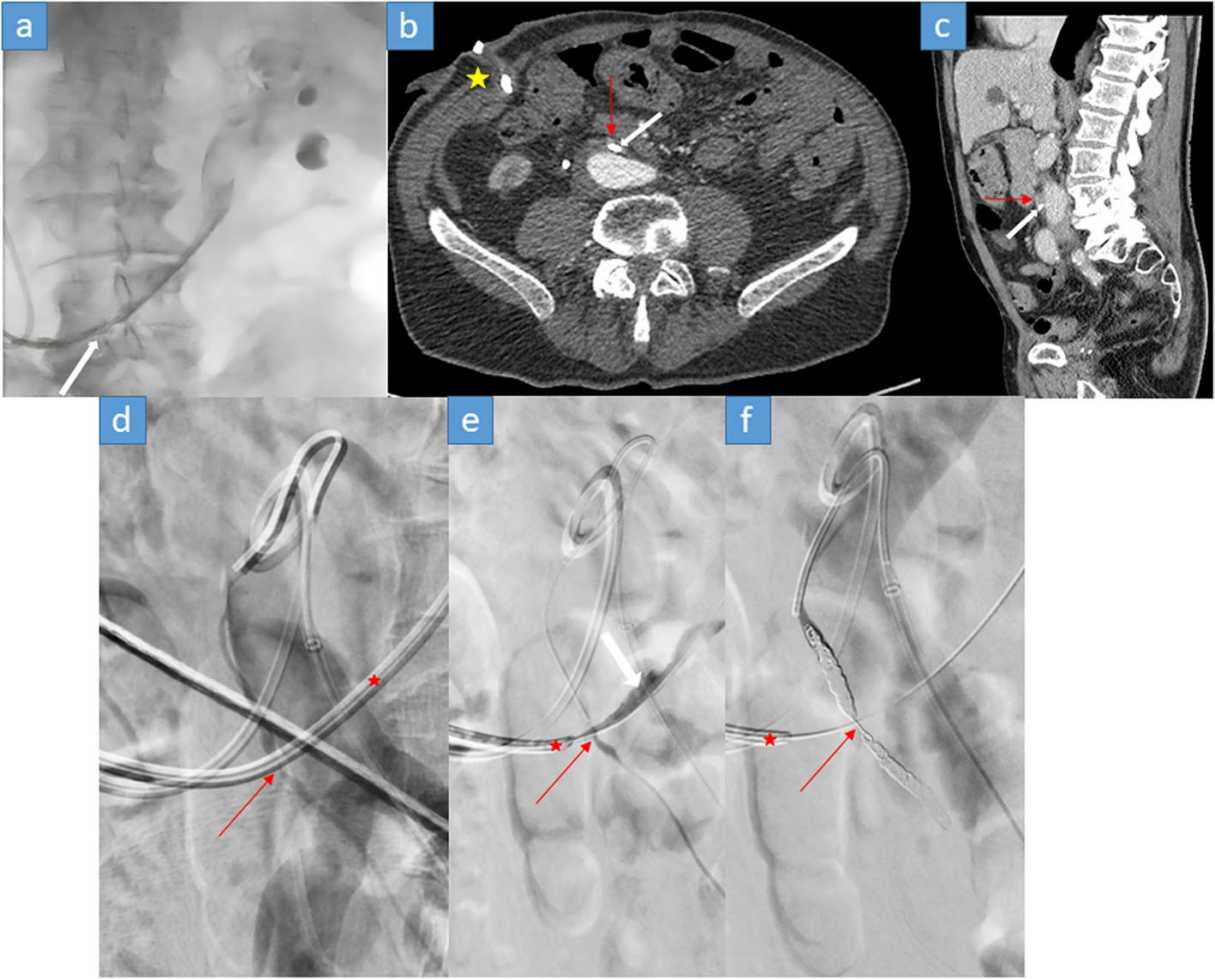
**a** R-UPG showing the flow from the ureter into a vascular structure (arrow). **b** and **c** A 2 months old CT scan showing the course of the left ureter (white arrow) crossing the midline between the aorta and the IMA (red arrow) after bilateral ureterocutaneostomy on the right side (star) with bilateral ureteral stents. **d** Overview DSA with an apparently normal IMA at the uretero-arterial crossing point (red arrow). Notice the lying left ureteral stent in place (star). **e** A selective DSA of the IMA using a microcatheter with direct visualization of contrast medium flowing into the ureter (white arrow) with a clear UVF at the uretero-arterial crossing point as direct visualization of the UVF (red arrow) after provocation by pulling the stent beyond the crossing point by the urologist (star). **f** Front door – back door coil embolization of the IMA with the starting point distal to the uretero-arterial crossing point (red arrow). Despite the stent remaining in a retracted position (star), complete elimination of the UVF through the coils was achieved

After radical prostatectomy and radiation therapy, our patient number 8 suffered from recurrent vesicointestinal fistulae. This was managed by cystectomy and ureterocutaneostomy on the right side with bilateral ureteral stents. The patient presented with recurrent macrohematuria and acute pain of the left flank. During an exchange of the left ureteral stent, the patient started bleeding massively after removal of the stent. An immediate tamponade of the bleeding and then a quick retrograde ureteropyelogaphy (R-UPG) was sufficient to see a connection to the arterial vascular system (Fig. 5a). A previous CT (2 months prior) showed the possible vascular contacts to the left ureter. Initially, it was believed to be the aorta due to the massive bleeding and due to the previous publications of aortic involvement in UVF (Fig. 5b). Immediately before the angiographic examination, a direct proximity of the ureter to the IMA was noticed and was considered as a possible vascular component of the UVF (Fig. 5c). The initial DSA was negative (Fig. 5d) and thereby a selective DSA of the IMA with provocation by partially pulling the catheter by the urologist before injecting contrast medium was performed, revealing the direct fistula’s connection (Fig. 5e). A front door – back door coil embolization of the IMA was performed to prevent any possible retrograde bleeding over the arc of Riolan successfully (Fig. 5f). Therefore, it is important to consider every vessel in proximity of the ureteral course as a possible vascular side of the UVF. A careful analysis of the cross-sectional imaging in this case was the key to avoid unnecessary implantation of an aortic stent graft, which would have made any future endovascular therapy of the case extremely difficult since the origin of the IMA would have been blocked by the stent graft. Another critical take home message to consider.

Limitation of the study was the dispute in some cases to whether the UVF location was the CIA or the EIA due to the anatomical fact that the ureter crosses the CIA immediately proximal to the iliac bifurcation. This dispute was settled in this review for the CIA, which would slightly increase the percentage attributed to the CIA at the expense of the EIA. In some other cases, UVF could not be directly visualized, but the location would still be considered and included as long as the main criteria such as vascular changes (e.g. irregular wall, stenosis, pseudoaneurysm, etc.) or direct contact to the vessel was provided through a sufficient imaging modality (usually CT angiography).

## Data Availability

Internal PACS system of Helios Klinikum Erfurt.
